# Electrical characterization of single molecule and Langmuir–Blodgett monomolecular films of a pyridine-terminated oligo(phenylene-ethynylene) derivative

**DOI:** 10.3762/bjnano.6.116

**Published:** 2015-05-11

**Authors:** Henrry Marcelo Osorio, Santiago Martín, María Carmen López, Santiago Marqués-González, Simon J Higgins, Richard J Nichols, Paul J Low, Pilar Cea

**Affiliations:** 1 Departamento de Química Física, Facultad de Ciencias, Universidad de Zaragoza, 50009 Zaragoza, Spain; 2 Instituto de Nanociencia de Aragón (INA), Edificio I+D, Campus Rio Ebro, Universidad de Zaragoza, C/Mariano Esquillor s/n, 50017 Zaragoza, Spain; 3 Laboratorio de Microscopias Avanzadas (LMA) C/Mariano Esquilor s/n, Campus Rio Ebro, 50018 Zaragoza, Spain; 4 Instituto de Ciencia de Materiales de Aragón (ICMA), Universidad de Zaragoza-CSIC, 50009 Zaragoza, Spain; 5 Department of Chemistry, University of Durham, Durham DH1 3LE, United Kingdom; 6 Department of Chemistry, University of Liverpool, Crown Street, Liverpool L69 7ZD, United Kingdom; 7 School of Chemistry and Biochemistry, University of Western Australia, Crawley 6009, WA, Australia

**Keywords:** Langmuir–Blodgett films, molecular electronics, STM touch-to-contact method

## Abstract

Monolayer Langmuir–Blodgett (LB) films of 1,4-bis(pyridin-4-ylethynyl)benzene (**1**) together with the “STM touch-to-contact” method have been used to study the nature of metal–monolayer–metal junctions in which the pyridyl group provides the contact at both molecule–surface interfaces. Surface pressure vs area per molecule isotherms and Brewster angle microscopy images indicate that **1** forms true monolayers at the air–water interface. LB films of **1** were fabricated by deposition of the Langmuir films onto solid supports resulting in monolayers with surface coverage of 0.98 × 10^−9^ mol·cm^−2^. The morphology of the LB films that incorporate compound **1** was studied using atomic force microscopy (AFM). AFM images indicate the formation of homogeneous, monomolecular films at a surface pressure of transference of 16 mN·m^−1^. The UV–vis spectra of the Langmuir and LB films reveal that **1** forms two dimensional J-aggregates. Scanning tunneling microscopy (STM), in particular the “STM touch-to-contact” method, was used to determine the electrical properties of LB films of **1**. From these STM studies symmetrical *I–V* curves were obtained. A junction conductance of 5.17 × 10^−5^ *G*_0_ results from the analysis of the pseudolinear (ohmic) region of the *I–V* curves. This value is higher than that of the conductance values of LB films of phenylene-ethynylene derivatives contacted by amines, thiols, carboxylate, trimethylsilylethynyl or acetylide groups. In addition, the single molecule *I*–*V* curve of **1** determined using the *I*(*s*) method is in good agreement with the *I–V* curve obtained for the LB film, and both curves fit well with the Simmons model. Together, these results not only indicate that the mechanism of transport through these metal–molecule–metal junctions is non-resonant tunneling, but that lateral interactions between molecules within the LB film do not strongly influence the molecule conductance. The results presented here complement earlier studies of single molecule conductance of **1** using STM-BJ methods, and support the growing evidence that the pyridyl group is an efficient and effective anchoring group in sandwiched metal–monolayer–metal junctions prepared under a number of different conditions.

## Introduction

Molecular electronics, in which a single molecule or a single layer of molecules is oriented between two electrodes to create a nascent device with the critical distance between the contacts in the nanometer size range [1–2], has potential to serve a role in the development of a new technology that could overcome the difficulties now being encountered during top-down scaling of conventional silicon technology. The advantages of the use of molecules as circuit elements include: a further reduction in the size of active components (and hence, a further increase in the density of devices), potentially cheaper devices through the increased use of self-assembly of complex structures, whilst quantum effects [3–6] may permit the appearance of new functions and technological applications not possible with conventional semiconductors such as quantum information processing [7], quantum computation [8], thermoelectric energy conversion [9], etc. The study of single-molecule junctions has enormously contributed to our ability to understand and control charge and heat transport phenomena at the molecular scale [10–21]. Complementary studies of larger area metal–molecular monolayer–metal junctions play a further crucial role in understanding the effect of intermolecular interactions, for example, van der Waals interactions and polarization effects in electronic transport properties [22–24]. In addition, planar-sandwiched monolayer structures are more closely aligned with practical electronic applications.

Three main techniques have been used to fabricate molecular assemblies for their study in the field of molecular electronics, namely, the self-assembly (SA), electrografting and Langmuir–Blodgett (LB) methodologies [25–29]. SA monolayers are easy to prepare and this method leads to highly ordered films. However, directionally oriented films of molecules containing two different groups, each capable of interacting with the substrate, cannot be prepared by this method [30]. Also, the molecule–substrate and molecule–molecule interactions required for the formation of robust, well-ordered SA films result in a rather limited number of metal–organic interfaces available to be studied [31–32]. Electrografted molecules form robust bonds with the underlying substrate but are typically not as well ordered as SA or LB films, and the growth of less defined multilayers is common with this method [33]. The LB technique requires a tedious fabrication process; however, this method provides many possibilities for the fabrication of well-ordered mono and multilayered films [34]. LB films also offer the possibility of exploration of a large number of metal–organic interfaces involving either physi- or chemi-sorbed films [31], and also permits the fabrication of directionally oriented monolayers when the molecule contains two different terminal groups that each have affinity for the substrate [30]. In particular, LB films have been used to analyze different properties and explore potential applications including molecular switching behavior [35–36], rectifying molecular junctions [37–38], exciton migration control [39], top-contact metallization [24,40–41], optical and opto-electronic applications [42–43], modulation of the electrical properties of the junction [24], inclusion of a metal atom in the organic structure of a molecular wire [44], and electrical measurements of both molecular ensembles and single molecules in the constrained environment of the film [24,30,45].

It is now well-known that charge transfer through metal–molecule–metal junctions is dependent not only on the molecular backbone but also on the metal–molecule contacts, and many functional groups have been studied in an attempt to find an ideal combination of molecular backbone, contact and metallic electrodes. Particularly prominent examples of metal–molecule contacting groups include thiols [46–47], selenols [48–49], dithiocarbamates [50–51], carbodithioates [52], amines [53–54], esters [55], cyano [56–57], isocyanides [58], nitriles [59], carboxylic acids [24,55,60], dithiocarboxylic acids [52], isothiocyanates [61], dimethylphosphine [62], 4-(methylthio)phenyl groups [63], dihydrobenzo[*b*]thiophenes [64], thienyl rings [65], diphenylphosphine group [66], trimethylsilylethynyl groups [67–69] and fullerenes [60,70–71]. However, many of these groups have significant limitations including chemical degradation at working temperatures [72–73], associated polymerization phenomena [74], small binding energies [74], unexpectedly high contact resistance [75–80], and multiple conductance values due to the variability in the binding geometries [81–86].

**Figure 1:**
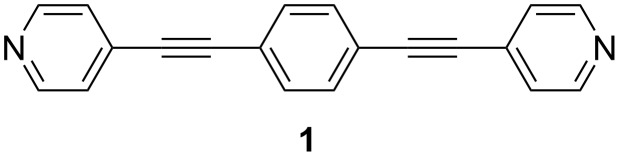
Chemical structure of 1,4-bis(pyridin-4-ylethynyl)benzene (**1**).

The chemical affinity of the pyridyl moiety for gold together with the strongly delocalized π system and chemical compatibility with a wide range of conjugated sub-structures commonly employed in molecular electronics have focused attention on this potential linker group as an alternative to solve these problems [87]. Previous studies of pyridyl-functionalized molecules in single molecule conductance studies [19,21,88–91] have revealed that this moiety can work as an anchoring group, forming stable and reproducible molecular junctions with relatively high conductance, and statistically high junction formation probabilities in the break junction method. In addition, the chemical inertness of the pyridyl group makes it quite attractive, since no protective groups are needed in the synthesis or deployment as a contact group (cf. thioacetate, –SAc, commonly used to prepare thiolate-contacted junctions). These promising features and results from single molecule studies have motivated us to explore the electrical properties of a monomolecular Langmuir–Blodgett film of **1** (Figure 1), and to draw comparisons with the single molecule conductance as well as with other monolayers containing phenylene-ethynylene derivatives incorporating different terminal groups. The results presented here reveal that the strong Au–N donor–acceptor (D–A) bond results in metal–monolayer–metal devices exhibiting a relatively high conductance.

## Results and Discussion

### Fabrication and characterization of Langmuir and Langmuir–Blodgett films

**Figure 2:**
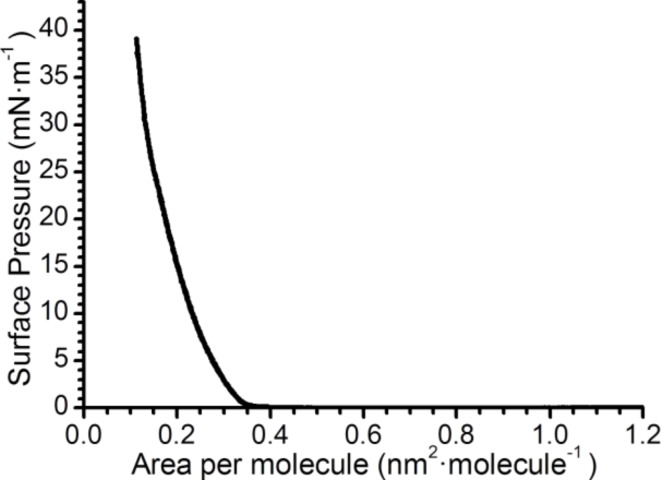
Surface pressure vs area per molecule isotherm of **1** at 20 °C.

**Figure 3:**
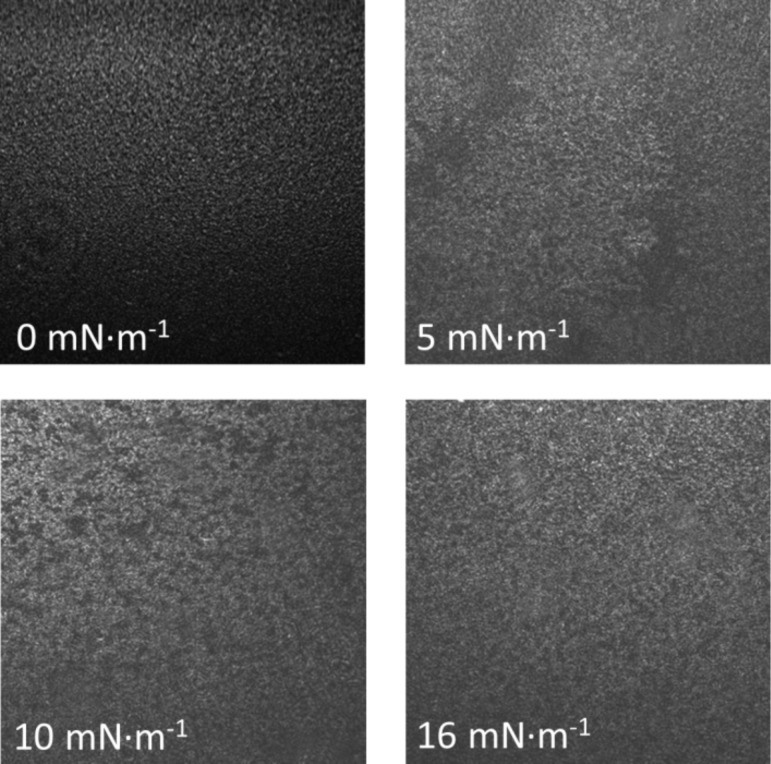
BAM images of **1** at the air–water interface at the indicated surface pressures.

Surface pressure–area per molecule (*π*–*A*) isotherms of **1** on a pure water subphase were recorded and reproducible results were obtained. One of these reproducible isotherms is illustrated in Figure 2. This isotherm shows a zero surface pressure in the 1.2–0.35 nm^2^·molecule^−1^ range, which corresponds to a monolayer in the gas phase. At 0.35 nm^2^·molecule^−1^ there is a lift-off in the π–*A* isotherm, which is followed by a monotonous increase of the surface pressure upon compression. In addition, Brewster angle microscopy (BAM) images were recorded at different stages of compression as illustrated in Figure 3, which reveal the formation of homogeneous films at the air–water interface. The BAM images exhibit an increase in the brightness upon compression which is indicative of a gradual tilt of the molecules towards alignment normal to the water surface. In addition, neither 3D aggregates nor crystals can be observed within the mini-BAM microscope resolution (<12 μm).

**Figure 4:**
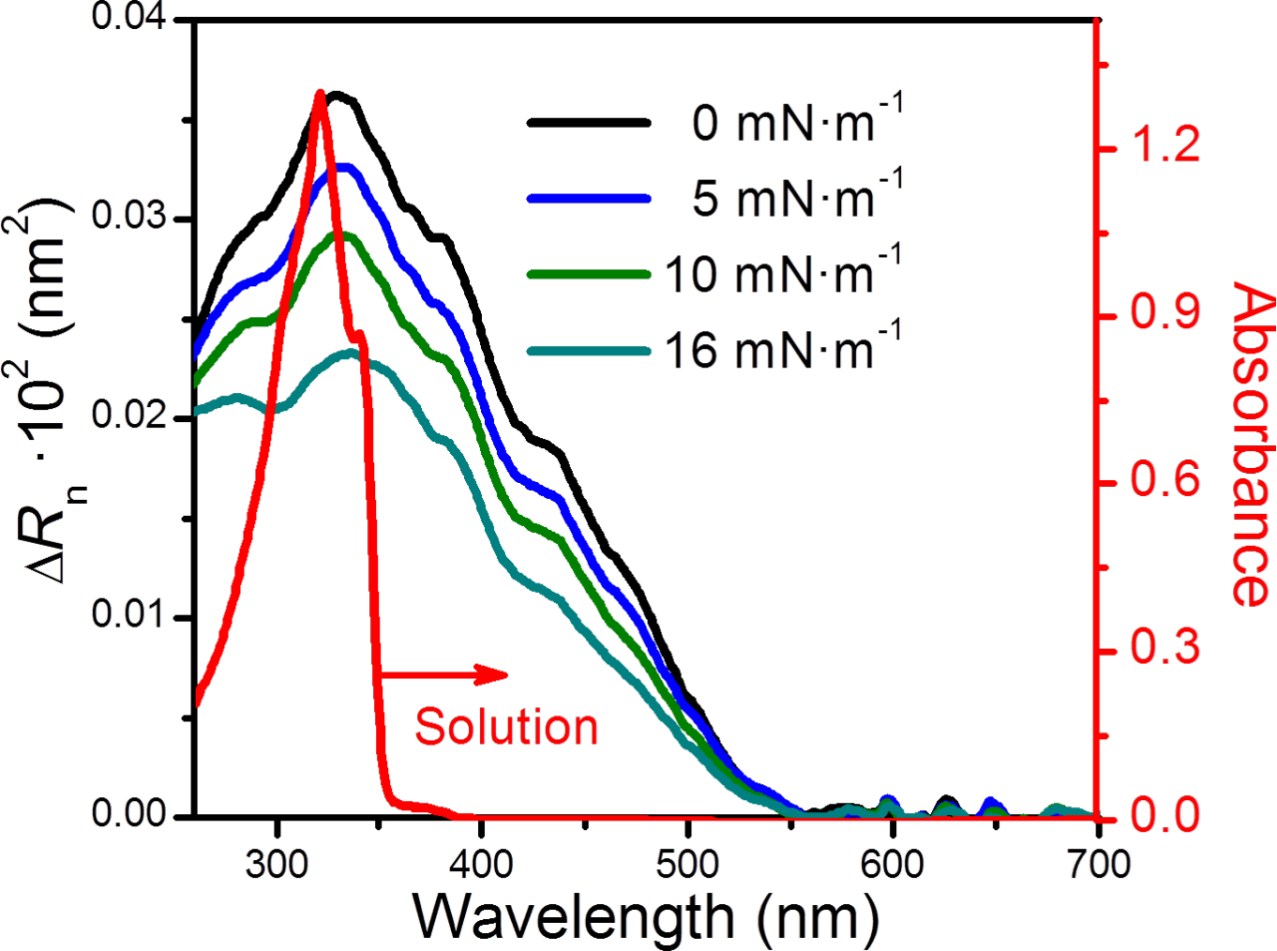
Normalized reflection spectra upon compression at the indicated surface pressure (left) and absorption spectrum of a 2.5 × 10^−5^ M solution of **1** in CHCl_3_ (right).

UV–vis reflection spectroscopy was used to complement the information obtained by the *π*–*A* isotherm and BAM images. Figure 4 shows the normalized reflection spectra, Δ*R*_norm_, of the Langmuir films of **1** (Δ*R*_norm_ = Δ*R*·*A*, where Δ*R* is the reflection and *A* is the area per molecule) for different values of the area per molecule. For comparison purposes, the UV–vis absorption spectrum of **1** (2.5 × 10^−5^ M) in CHCl_3_ is also shown. The broad absorption spectra and the red shift of the absorption edge indicate the presence of various two dimensional (2D) J-aggregates of **1** on the water surface [92–93]. J-aggregates, named after E. E. Jelley who first discovered them [94], are formed by molecules arranged in an edge-to-edge configuration and characterized by an absorption band shifted to a longer wavelength compared to the monomer. Langmuir films of **1** show a decrease in the Δ*R*_n_ values upon compression, which indicates that there is a gradual decrease of the tilt angle formed by the normal to the surface and the dipole transition moment of the chromophore. This result is in agreement with a progressive reorientation of the molecules in the Langmuir film upon compression.

**Figure 5:**
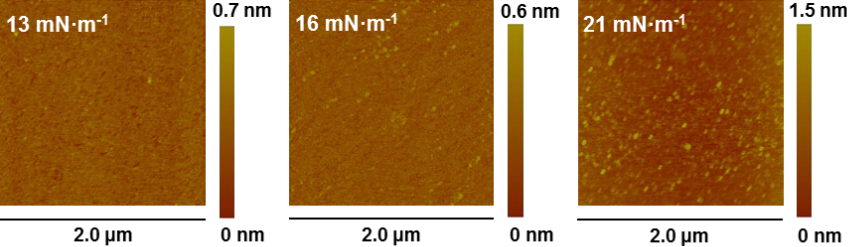
AFM images of a single-layer LB film of **1** transferred onto freshly cleaved mica at the indicated surface pressure.

Langmuir–Blodgett monomolecular films of **1** were obtained by the transference of Langmuir films onto solid substrates by the vertical dipping method during the upstroke of hydrophilic substrates initially immersed in the subphase. Monolayers of **1** were deposited onto freshly cleaved mica substrates at different transfer surface pressures in order to determine their homogeneity and quality by means of atomic force microscopy (AFM). The final aim of this AFM study was to find the optimum surface pressure of transference. Figure 5 shows AFM images of Langmuir–Blodgett films of **1** transferred at 13, 16 and 21 mN·m^−1^. These images show mica substrates practically covered by the monolayer. AFM images of films transferred at a surface pressure of 21 mN·m^−1^ exhibit a root mean squared (RMS) surface roughness of 0.197 nm and indicate less homogeneous monolayers. In contrast, the film roughness was 0.145 nm and 0.098 nm at 13 mN·m^−1^ and 16 mN·m^−1^, respectively, indicating that the optimum surface pressure of transference is 16 mN·m^−1^. At this surface pressure of transference, an LB film free of holes and three dimensional (3D) defects is obtained.

The deposition ratio of the monolayer onto a solid substrate is defined as the decrease in the monolayer area during the transfer process divided by the area of the substrate. The deposition ratio of the monolayer during the upstroke of the film transfer process was determined by the software controlling the Langmuir trough, resulting in a value close to unity for a surface pressure of transference of 16 mN·m^−1^. This high deposition ratio was also demonstrated using a quartz crystal microbalance (QCM). The frequency change (Δƒ) for a QCM quartz resonator before and after the deposition process was experimentally determined. This frequency change can be introduced in the Sauerbrey equation [95]:

[1]
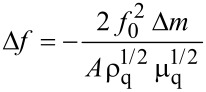


to determine the surface coverage. In Equation 1, *f*_0_ is the fundamental resonance frequency of 5 MHz, Δ*m*(g) is the mass change, *A* is the electrode area, ρ_q_ is the density of the quartz (2.65 g·cm^-3^), μ_q_ is the shear modulus (2.95 × 10^11^ dyn·cm^−2^), and the molecular weight of **1** is 280 g·mol^−1^. Thus, the surface coverage of **1** incorporated into LB films, obtained from Equation 1, is 0.98 × 10^−9^ mol·cm^−2^. This value is in excellent agreement with the estimated value determined from the molecular area of **1**, which is 1.01 × 10^−9^ mol·cm^−2^ at a surface pressure of 16 mN·m^−1^.

**Figure 6:**
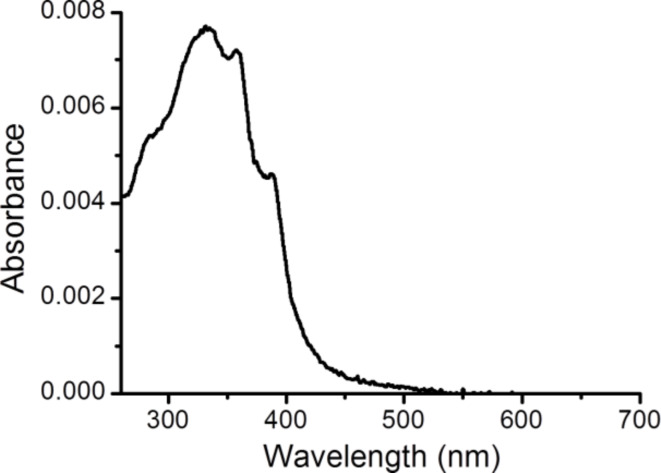
Absorbance of a monomolecular Langmuir–Blodgett film of **1** transferred at 16 mN·m^−1^ onto a quartz substrate during the withdrawal of the substrate from the water subphase.

The UV–vis absorption spectrum of the LB film of **1** transferred onto quartz substrates at 16 mN·m^−1^ during the upstroke of the substrate was recorded (Figure 6) in order to obtain additional information about the molecular arrangement of **1** in LB films. The spectrum exhibits a similar profile to the reflection spectrum of the Langmuir film at 16 mN·m^−1^, and is characterized by a maximum absorption feature at 335 nm and a broad band with several shoulders, indicating again the presence of lateral 2D J-aggregates.

### Electrical properties of LB films of 1

**Figure 7:**
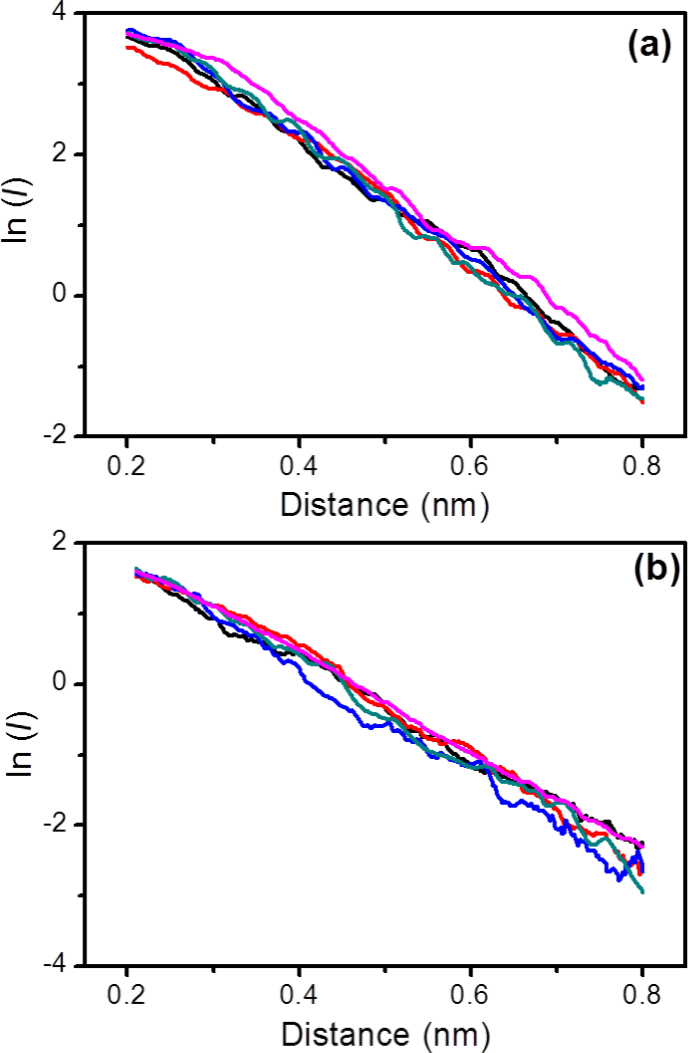
(a) ln *I* vs *s* plots used for the calibration of tip–substrate distance (a) for **1** in LB films (for recording dlnI/ds for inside the LB film the tip was retracted from deep in the LB film to the length of the vertically extended molecule, with dlnI/ds remaining relatively low over this range); and (b) for **1** single molecule.

The electrical properties of monomolecular LB films of **1** deposited on Au(111) as described above were studied using a scanning tunneling microscope (STM) and the “STM touch-to-contact” method [23–24,30,44–45]. The “STM touch-to-contact” method requires the STM tip to be positioned immediately above and just touching the LB film, avoiding both penetration of the STM tip into the film or a significant gap between the STM tip and the monolayer. This in turn requires calibration of the tip–substrate separation as well as an accurate, independent determination of the LB film thickness. The thickness of the monolayer (1.70 ± 0.05 nm) was determined using the attenuation of the Au 4f signal in the XPS spectra as described in the Experimental section. The calibration of the tip–substrate distance was carried out by relating the STM set-point parameters (set point current, *I*_0_, and tip bias, *U*_t_) to an absolute tip-to-substrate separation as previously described [30,44–45,77,96–97]. Current–distance retraction scans (*I*(*s*) curves) were recorded by first setting the STM tunneling parameters (*I*_0_ = 60 nA and *U*_t_ = 0.6 V) so that the tip approaches relatively close to the surface and is thereby embedded within the LB film. From these set-point conditions the STM tip was then rapidly retracted while monitoring the current decay with distance. Only current–distance retraction traces that displayed a monotonic exponential decrease of the tunneling current (no wire formation) were selected for estimation of the distance decay of the current within the LB film as quantified by the dln*I*/d*s* value, as described below. These calibration data were recorded separately during the jump-to-contact measurements at regular time intervals and at different substrate locations. The collected calibration *I*(*s*) curves were plotted as linear ln*I* vs *s* plots. Figure 7a shows five overlaid ln*I* vs *s* curves measured on **1** LB films. The nonlinear region at the beginning of the ln*I* vs distance curve has been omitted (this was attributed to an initial inertia in the retraction process, caused by an initial piezo delay). Linear regression was then used to determine the slope of the ln*I* vs *s* plots, with typical slopes of 5.80 ± 1.06 nm^−1^. This value is in good agreement with those reported for similar molecular films of highly conjugated organic compounds [23–24,30,45,98–99] and for single molecules [15,100–101].

The dln*I*/d*s* value for the LB film is used in conjunction with Equation 2 and an extrapolation to the conductance value corresponding to the point where the gold STM tip contacts the gold substrate (taken as *G*_0_ where *G*_0_ = 2*e*^2^/*h* = 77.4 μS) to estimate the current and voltage set-point values where the STM tip would touch the top of the LB monolayer film. Taking the measured dln*I*/d*s* value of 5.80 nm^−1^, the set-point parameters *I*_0_ = 2.5 nA and *U*_t_ = 0.6 V yield a tip–substrate distance estimation of 1.70 nm, which corresponds to the independently determined thickness of the monolayer. Therefore, using these set-point conditions, *I–V* curves can be recorded with the STM tip directly in contact with the top of the LB monolayer. If another set-point parameter is chosen so that the tip is embedded within the LB film, the dln*I*/d*s* values and Equation 2 could be used to estimate the distance of the tip within the film. In contrast, if a set-point parameter is chosen so that the tip is above the top of the LB film, then the dln*I*/d*s* cannot be used to estimate the position of the tip, since these dln*I*/d*s* values are different from those within the film.

[2]
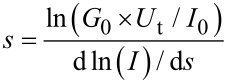


**Figure 8:**
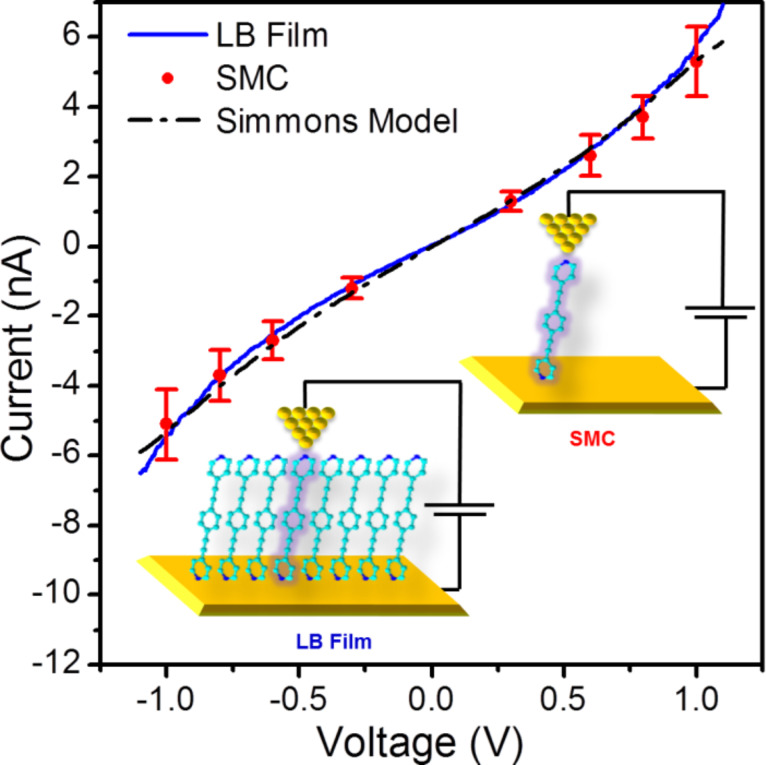
*I–V* curve of a single layer LB film of **1** transferred onto Au(111) at a surface pressure of 16 mN·m^−1^ using *I*_0_ = 2.5 nA and *U*_t_ = 0.6 V as set-point parameters (blue line) and fitted according to the Simmons equation using φ = 0.71 eV, α = 0.35 (black dashed line). An *I–V* curve constructed from single molecule conductance values obtained using the *I*(*s*) method is also shown (red circles). The error bars represent the standard deviation obtained from the widths of the conductance histogram peaks.

Using these “touch-to-contact” set-point parameters (*I*_0_ = 2.5 nA and *U*_t_ = 0.6 V), over 300 current–voltage (*I–V*) curves were recorded from different substrates and at different substrate locations and averaged to ensure the reproducibility and reliability of the results. Figure 8 shows a representative *I–V* curve obtained for a single layer LB film transferred onto Au(111) at a surface pressure of 16 mN·m^−1^ and recorded under touch-to-contact conditions. The profile of the *I*–*V* curve is clearly symmetrical around zero bias and exhibits a characteristically curved shape over the full bias voltage region spanning between −1 V to +1 V. In the low-voltage region (from −0.5 to +0.5 V), the *I*–*V* curve is relatively linear, and from this “ohmic” region, a conductance of 5.17 × 10^−5^ *G*_0_ is obtained.

Some important parameters relating to the conductance of films of **1** and closely related compounds are given in Table 1. Although a rigorous quantitative comparison cannot be made between the full series of molecules in Table 1 due to differences in LB film thickness (monolayer LB films of the shortest molecule **1** being thinner than the other LB films), the conductance value for compound **1** is 3–20 times greater than for LB films of other oligo(phenylene-ethynylene) (OPE) derivatives bearing anchoring groups such as thiol (–SH), amine (–NH_2_), carboxylate (–COO^−^), trimethylsilylethynyl (–C≡CSiMe_3_) or acetylide (–C≡C) [23–24,30,45]. Similar variations in conductance as a function of surface contacting group have been found for polymethylene (alkane) bridges contacted with thiol, amine or carboxylic acid moieties to gold electrodes [75]. The higher conductance for **1** could be attributed to both the shorter molecular length and efficient pyridyl–Au contacts. Previous contributions in the field have shown that the charge transport in molecular wires incorporating electron-withdrawing pyridyl-type anchoring groups is preferentially controlled by the lowest unoccupied molecular orbital (LUMO). That is, the pyridyl group decreases the frontier orbital energies and promotes electron transport by reducing the energy offset between the molecular LUMO and the Fermi level of electrodes [102–104]. In particular, DFT-based studies of **1** in single molecule junctions have shown that the total conductance is controlled by eigenchannels consisting of the molecular π* LUMO coupled to Au p states at the binding site [105]. In addition, the direct N–Au (D–A) bond between the highly conjugated molecular structure of **1** and the metal electrode [74] avoids any non-conjugated spacer groups.

**Table 1:** Conductance values for the listed OPE compounds incorporated in monolayer LB films determined by using the “STM touch-to-contact” method. The length of the molecule, together with the monolayer thickness (which is a function of the molecule length, and the tilt angle of the molecule with respect to the substrate surface), are also indicated.

Compound	Molecule length (nm)	Monolayer thickness (nm)	Conductance	Reference

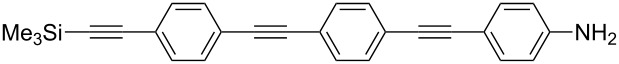	2.23	1.49 ± 0.04	1.20 × 10^−5^ *G*_0_	[23]
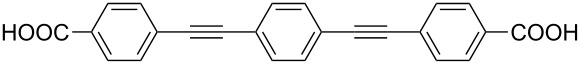	2.07	1.81 ± 0.05	0.26 × 10^−5^ *G*_0_^a^ 1.75 × 10^−5^ *G*_0_^b^	[24]
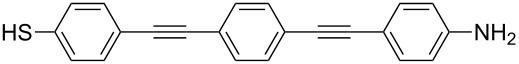	2.03	1.77 ± 0.05	1.37 × 10^−5^ *G*_0_	[30]
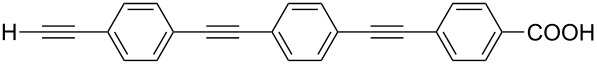	2.12	2.01 ± 0.05	1.48 × 10^−5^ *G*_0_	[45]
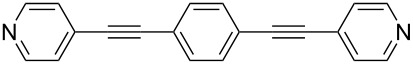	1.64	1.70 ± 0.05	5.17 × 10^−5^ *G*_0_	This work

^a^Compound linked through a deprotonated carboxylic group to the gold substrate and a carboxylic acid group (forming H bonds with neighboring molecules) to the STM tip. ^b^Compound linked through deprotonated carboxylic groups to the gold substrate and the STM tip.

In single molecule conductance studies, conjugated molecules similar to **1** with two pyridyl terminal groups exhibit two conductance values, which have been attributed to two distinct binding geometries in the molecular junction [105]. The lower of these two conductance values has been assigned to the simplest N–Au binding of the molecular normal to a flat metal surface or terrace, that is, the distance between the electrodes is directly related to the length of the molecule. The higher conductance value has been attributed to a tilted configuration that gives increased coupling between the π system of the pyridyl ring and the gold surface [105]. In contrast, compound **1** only shows one conductance value when it is arranged in an LB film, which corresponds to the lower of the two conductance values measured in single molecule junctions [88,105]. This unique conductance value may be induced by the constrained molecular orientation of **1** in a well-ordered and highly packed monomolecular LB film, where the molecules are arranged in a rather upright orientation with respect to the bottom electrode.

**Figure 9:**
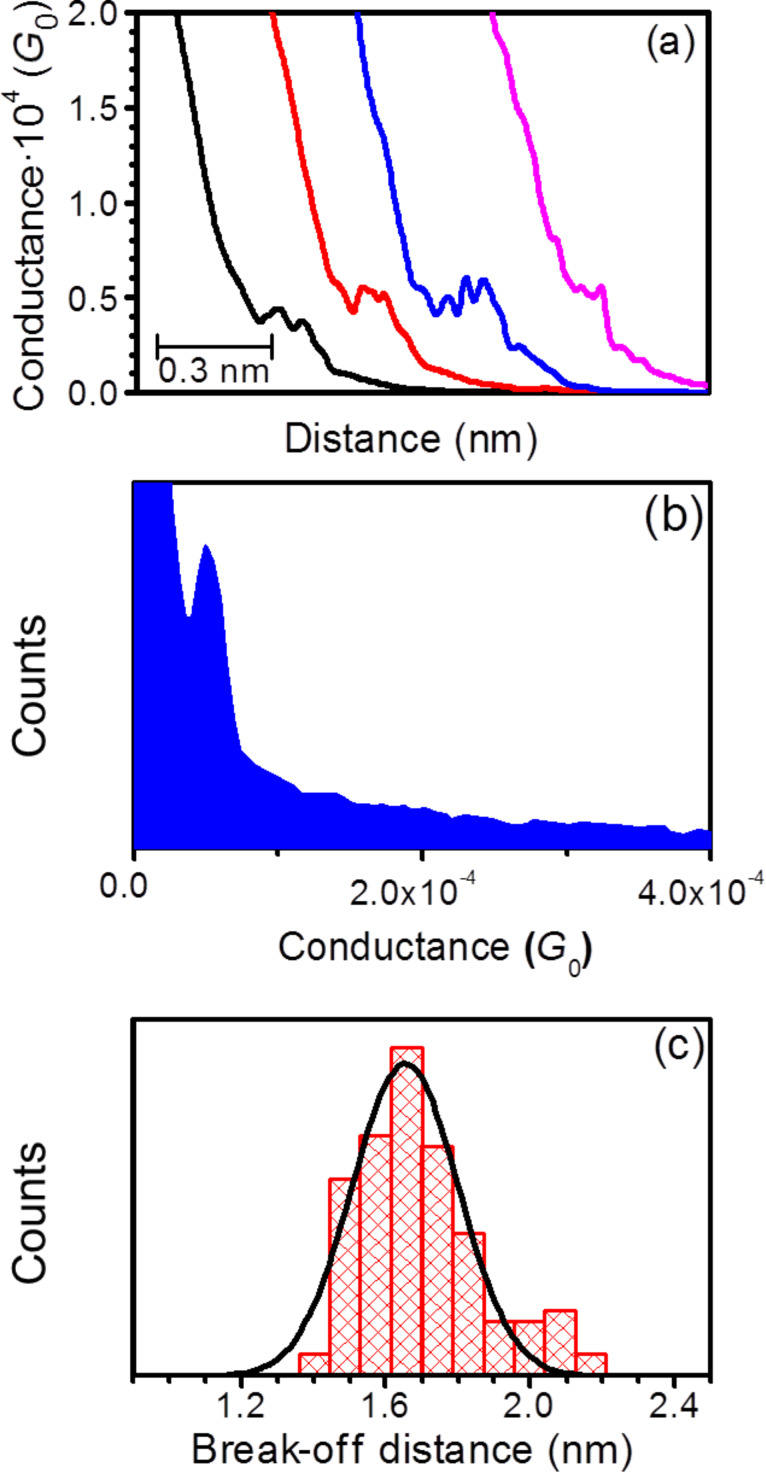
(a) Typical conductance traces of **1** using the *I*(*s*) method. The curves have been shifted horizontally for clarity. (b) Conductance histogram built by adding together all the points of 300 conductance traces that show discernible plateaus such as those displayed in (a). (c) Break-off distance histogram. Conductance data are presented in units of the conductance quantum (*G*_0_ = 2*e*^2^/*h* = 77.4 μS), *U*_t_ = −0.3 V.

Figure 8 also shows an *I*–*V* curve constructed from single molecule conductance (SMC) values for **1** obtained by using the *I*(*s*) method at eight different bias voltage values. The *I*(*s*) method developed by Haiss et al. has been widely used to determine the single-molecule conductance of different types of molecular bridges [77,88,97]. A detailed description of this method can be found in the literature [77,106–107] and in the Experimental section of this paper. *I*(*s*) curves, such as those shown as an example in Figure 9a at *I*_0_ = 10 nA and *U*_t_ = −0.3 V, were statistically analyzed in the form of a conductance histogram plot to determine the molecule conductance for a single molecule at the eight different bias voltage values as illustrated in Figure 9b. These conductance histograms were built by adding all the current (or conductance) points from approximately 300 current versus distance curves exhibiting a discernible plateau such as those shown in Figure 9a. In addition, a break-off distance histogram for **1** is shown in Figure 9c (corrected for the initial tip−substrate distance at the start of the *I*(*s*) scan according to Equation 2 with the selected set-point parameters for each bias and using a dln*I*/d*s* value of 7.0 ± 0.8 nm^−1^, which was determined in a similar manner to the one obtained for the LB film, Figure 7b). Therefore, the break-off distance refers to the estimated separation at which the molecular junction cleaves during an *I*(*s*) retraction experiment and it can be compared to the length of the molecule. The break-off distance obtained from Figure 9c (1.65 ± 0.2 nm) is in good agreement with the length of the molecule (1.64 nm) determined with a molecular modeling program (Spartan^®^08 V1.0.0). Meanwhile, the results obtained here for the SMC values of **1** (5.39 × 10^−5^ *G*_0_), which have been measured using the *I*(*s*) method and therefore correspond to the lower conductance value [88], are in good agreement with those published previously by Zhao et al. [108] who reported a conductance of 3.16 × 10^−5^ *G*_0_ for **1** using the mechanically controlled break junction method (MCBJ). The *I–V* curve determined for the LB film at 2.5 nA and 0.6 V is in excellent agreement with the SMC value of **1** obtained by means of the *I*(*s*) method. This result indicates that if these parameters are employed then the STM tip is located directly above the monolayer and also that the tip is electronically coupled to a single molecule. The similarity between the *I*–*V* curves obtained for the monomolecular LB film and for single molecules is of particular interest since the molecular environment is different in both cases. Whilst the molecules are closely packed within the LB film, no nearest molecules exist for the single molecule studies.

A widely applied tunneling model for non-resonant tunneling charge transport was developed by Simmons [109]. In this model, the current *I* is given by Equation 3:

[3]
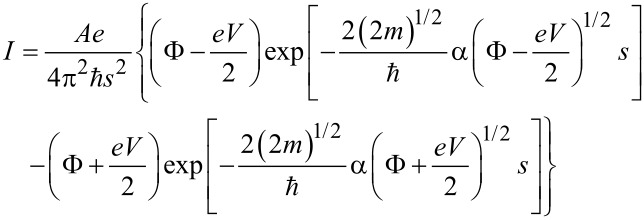


where *A* represents the contact area of the molecule with the gold surface (this value has been taken as 0.2 nm^2^ in concordance with the surface pressure vs area per molecule isotherm at a surface pressure of 16 mN∙m^−1^); *V* is the applied potential; *s* is the width of the tunneling barrier, which has been taken as 1.64 nm (value obtained from the geometric N…N distance determined with the molecular modeling program Spartan^®^08 V 1.0.0); φ represents the effective barrier height of the tunneling junction (relative to the Fermi level of Au); α is a fitting parameter related to the effective mass of the electron (or hole) when tunneling through the barrier; *m* and *e* represent the mass and the charge of an electron, respectively. Φ and α are the numerical parameters employed to fit the *I*–*V* data in Figure 8. In this work we used φ = 0.71 eV and α = 0.35, which lead to a good agreement between the experimental data and the model. Since Equation 3 fits our *I*–*V* data well, the mechanism of transport through these metal–molecule–metal junctions can be assumed to be nonresonant tunneling.

These collected electrical measurements indicate that the pyridyl group is an effective anchoring group in metal–molecule–metal and metal–monolayer–metal junctions formed by LB methods. The data indicate that it exhibits a higher conductance when compared with other anchoring end groups used in OPE derivatives assembled by the LB technique such as thiol, amine, carboxylic acid, trimethylsilylethynyl or acetylene.

## Conclusion

In this paper, a “symmetric” OPE derivative, with a pyridine group at both termini of the molecule has been synthesized and assembled by the Langmuir–Blodgett technique into well-packed monolayer films. Langmuir films were prepared at the air–water interface and characterized by *π*–*A* and Brewster angle microscopy, which revealed that this molecule can form true monomolecular films at the air–water interface. Atomic force microscopy images of LB films transferred at a surface pressure of 16 mN·m^−1^ revealed homogeneous films. QCM experiments demonstrated that monomolecular films of **1** were transferred onto solid substrates with a transfer ratio close to 1 and the UV–vis spectrum of the LB films shows the presence of 2D lateral molecular aggregates in a similar arrangement to that observed in the Langmuir films of **1**. Electrical characteristics of LB films deposited on gold substrates were studied using STM. The shape of the *I–V* curves and good fit with the Simmons model indicate that charge transport across of the metal–monolayer–metal junctions follows a nonresonant tunneling mechanism. Importantly, the conductance value in LB films (5.17 × 10^−5^ *G*_0_) is similar to the single molecule conductance values (5.39 × 10^−5^ *G*_0_ and 3.16 × 10^−5^ *G*_0_ when the *I*(*s*) method or the MCBJ was used, respectively), indicating that the conductance across to the molecule is not significantly influenced by the presence of neighboring *π* systems. Additionally, the obtained conductance value in LB films is higher than the values of monomolecular LB films of OPE derivatives containing other anchoring groups (thiol, amine, carboxylic acid, trimethylsilylethynyl or acetylene).

## Experimental

### Synthesis

**General conditions.** All reactions were carried out in oven-dried glassware under an oxygen-free nitrogen atmosphere using standard Schlenk techniques. Triethylamine was dried over CaSO_4_ and distilled and degassed before use. The catalyst Pd(PPh_3_)_4_ [110] and 1,4-diethynylbenzene [111] were prepared following literature methods. Other reagents were purchased commercially and used as received. The NMR spectra were recorded in deuterated solvent solutions on a Bruker Avance 400 spectrometer and referenced against solvent resonances. The ASAP mass spectra were recorded from solid aliquots on a Xevo QToF mass spectrometer (Waters Ltd., UK) in which the aliquot was vaporized using hot N_2_, ionized by a corona discharge and carried to the TOF detector (working range 100–1000 *m*/*z*).

**Scheme 1:**
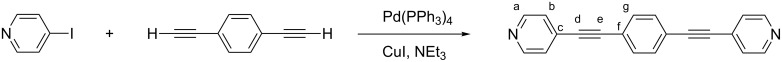
Preparation of 1,4-bis(pyridin-4-ylethynyl)benzene [108].

**Preparation of 1,4-bis(pyridin-4-ylethynyl)benzene,** Scheme 1 [108]. To a 100 mL Schlenk flask charged with NEt_3_ (100 mL), 4-iodopyridine (0.334 g, 1.63 mmol), 1,4-diethynylbenzene (0.101 g, 0.801 mmol), Pd(PPh_3_)_4_ (0.045 g, 0.039 mmol) and CuI (0.008 g, 0.042 mmol) were added. The suspension was stirred overnight at room temperature. The mixture was filtered and the colorless filtrate taken to dryness. The off-white solids were dissolved in Et_2_O (100 mL). The addition of trifluoroacetic acid generated a precipitate that was collected by filtration, washed thoroughly with Et_2_O and dried in air. The solids were redissolved in CH_2_Cl_2_ (25 mL) and extracted with aqueous KOH (0.1 M, 1 × 25 mL), water (1 × 25 mL) and brine (1 × 25 mL). The organic phase was collected, dried over MgSO_4_ and taken to dryness. The pure product was obtained as an off-white powder. The yield was 0.156 g, 0.556 mmol, 69%. ^1^H NMR (400 MHz, CDCl_3_) δ 8.62 (d, *J* = 5 Hz, 4H, *a*); 7.56 (s, 4H, *g*), 7.38 (d, *J* = 5 Hz, 4H, *b*). ^13^C NMR (101 MHz, CDCl_3_) δ 150.0 (*a*), 132.1(*g*), 131.2 (*c*), 125.6 (*b*), 123.0 (*f*), 93.3, 89.0 (*d/e*) [112]; MS(ASAP) *m/z* (%): 281.17 (100, [M + H]^+^).

### Film fabrication and characterization

LB films of **1** were prepared in a similar manner to other LB films incorporating oligo(phenylene-ethynylene) derivatives [28,44–45,55,113]. In particular, a Nima Teflon trough with dimensions 720 × 100 mm^2^, which was housed in a constant temperature (20 ± 1 °C) clean room, was employed to prepare the Langmuir films. A Wilhelmy paper plate pressure sensor was used to measure the surface pressure (π) of the monolayers. The subphase was pure water (Millipore Milli-Q, resistivity 18.2 MΩ·cm). A 2.5 × 10^−5^ M solution of **1** in CHCl_3_ (solvent purchased from LAB-SCAN Analytical Sciences and used as received; purity HPLC grade >99%) was spread onto the aqueous surface. The spreading solvent was allowed to completely evaporate over a period of at least 15 min before compression of the monolayer commenced at a constant sweeping speed of 0.015 nm^2^·molecule^−1^·min^−1^. Under these experimental conditions, the isotherms were highly reproducible. A commercial mini-Brewster angle microscope (mini-BAM) from Nanofilm Technologie GmbH, Göttingen, Germany, was employed for the direct visualization of the monolayers at the air–water interface and a commercial UV–vis reflection spectrophotometer (details described elsewhere [114]) was used to obtain the reflection spectra of the Langmuir films during the compression process.

The solid substrates used for the transfer were carefully cleaned as described elsewhere [115–116]. The monolayers were deposited onto several substrates (cleaved mica, gold and quartz) at a constant surface pressure of 16 mN∙m^−1^ by the vertical dipping method at a speed of 3 mm·min^−1^. UV–vis spectra were acquired on a Varian Cary 50 spectrophotometer and recorded at a normal incidence angle with respect to the film plane. AFM experiments employed to study the topography of the monolayers were performed by means of a Multimode 8 AFM system from Veeco, using tapping mode. The data were collected with a scan rate of 1 Hz and in ambient air conditions by using a silicon cantilever provided by Bruker, with a force constant of 40 N·m^−1^ and operating at a resonance frequency of 300 kHz.

X-ray photoelectron spectroscopy (XPS) spectra were acquired on a Kratos AXIS Ultra DLD spectrometer with a monochromatic Al Kα X-ray source (1486.6 eV) using a pass energy of 20 eV. The photoelectron take-off angle was 90° with respect to the sample plane. To provide a precise energy calibration, the XPS binding energies were referenced to the C 1s peak at 284.6 eV. The thickness of LB films on the gold substrates was estimated using the attenuation of the Au 4f signal from the substrate according to *I*_LB film_ = *I*_substrate_ exp(−*d*/λsinθ), where *d* is the film thickness, *I*_LB film_ and *I*_substrate_ are the average of the intensities of the Au 4f5/2 and Au 4f7/2 peaks attenuated by the LB film and from bare gold, respectively, θ is the photoelectron take-off angle, and λ is the effective attenuation length of the photoelectron (4.2 ± 0.1 nm) [117]. The QCM measurements were carried out using a Stanford Research System instrument and employing AT-cut, α-quartz crystals with a resonance frequency of 5 MHz having circular gold electrodes patterned on both sides.

An Agilent 5500 SPM microscope was used for characterization of the electrical properties of the LB films by recording the current, *I*, as a function of tip potential, *U*_t_. The STM tips were freshly prepared for each experiment by etching of a 0.25 mm Au wire (99.99%) in a mixture of HCl (50%) and ethanol (50%) at +2.4 V. The gold films were flame-annealed at approximately 800–1000 °C with a Bunsen burner immediately prior to use. This procedure is known to result in atomically flat Au(111) terraces [118].

The *I*(*s*) method was used to determine the single molecule conductance values of **1***.* For a given set-point current and bias voltage, typically 3,500–4,000 events were observed, but only those curves showing current steps associated with the formation of molecular bridges were recorded, that is, approximately 300 at each different bias voltage value. These curves were then statistically analysed in the form of histogram plots to determine the single molecule conductance. Molecular adsorption was achieved by immersion of **1** solution in CHCl_3_ (0.1 mM) for about 60 s. After adsorption, the sample was washed in ethanol and then blown dry in a stream of nitrogen. All *I*(*s*) measurements were conducted in mesitylene.
